# Whole Genome-Based Characterization of Multidrug Resistant Enterobacter and Klebsiella aerogenes Isolates from Lebanon

**DOI:** 10.1128/spectrum.02917-22

**Published:** 2023-01-18

**Authors:** Georgi Merhi, Sara Amayri, Ibrahim Bitar, George F. Araj, Sima Tokajian

**Affiliations:** a Department of Natural Sciences, Lebanese American University, Byblos, Lebanon; b Department of Microbiology, Faculty of Medicine, and University Hospital in Pilsen, Charles University, Pilsen, Czech Republic; c Biomedical Center, Faculty of Medicine in Pilsen, Charles University, Pilsen, Czech Republic; d Department of Pathology & Laboratory Medicine, American University of Beirut Medical Center, Beirut, Lebanon; University of Guelph

**Keywords:** ESBL, *Enterobacter*, *Klebsiella aerogenes*, β-lactamases, multidrug resistance

## Abstract

Enterobacter spp. and Klebsiella aerogenes are rod-shaped Gram-negative opportunistic pathogens. This study aimed at the molecular and genomic characterization of multidrug resistant Enterobacter spp. and K. aerogenes isolates recovered from hospitalized patients in a tertiary care hospital in Lebanon. A total of 59 Enterobacter spp. clinical isolates consisting of 41 carbapenem-resistant and 18 susceptible by Etest were included in this study. Genotypic identification through whole-genome sequencing (WGS) was performed and confirmed *in silico*. Resistance and plasmid profiles were studied using ResFinder4.0 and Plasmid-Finder2.1. Multilocus sequence typing (MLST) was used to determine the isolates’ clonality. Using the average nucleotide identity (ANI) we identified and confirmed that 47 (80%) isolates were *E*. *hormaechei*, 11 (18%) were Klebsiella aerogenes and 1 (2%) was an E. cloacae. Carbapenem-resistance was detected among 41 isolates all showing an MIC_90_ of ≥ 32 μg/mL for ertapenem, imipenem, and meropenem. *bla*_NDM-1_ (58.5%), *bla*_ACT-16_ (54%), and *bla*_OXA-1_ (54%) were the most common detected β-lactamases, while *bla*_CTX-M-15_ (68%) was the main detected extended-spectrum β-lactamase (ESBL) encoding gene. Chromosomal *ampC*, carbapenemase encoding genes, and porin modifications were among the detected carbapenem resistance determinants. The carbapenemase encoding genes were linked to three well-defined plasmid Inc groups, IncFII/IncFIB, IncX3, and IncL. MLST typing revealed the diversity within the studied isolates, with ST114 being the most common among the studied *E*. *hormaechei.***:** The spread of carbapenem-resistant isolates in clinical settings in Lebanon is a serious challenge. Screening and continuous monitoring through WGS analysis could effectively limit the dissemination of drug-resistant isolates in hospitalized patients.

**IMPORTANCE** Drug resistance is an increasing global public health threat that involves most disease-causing organisms and antimicrobial drugs. Drug-resistant organisms spread in health care settings, and resistance to multiple drugs is common. Our study demonstrated the mechanisms leading to resistance against the last resort antimicrobial agents among members of the *Enterobacteriaceae* family. The spread of carbapenem-resistant bacteria in clinical settings is a serious challenge. Screening and continuous monitoring could effectively limit the dissemination of drug-resistant isolates in hospitalized patients.

## INTRODUCTION

Enterobacter cloacae complex (ECC) members and Klebsiella aerogenes belong to the family *Enterobacteriaceae* and are Gram-negative, rod-shaped, nonspore forming opportunistic pathogens causing health care-associated infections (HAIs) (1–3). ECC members are saprophytic microorganisms in that they inhabit and colonize diverse environments such as sewage, soil, and the human GI tract (4). Detection of K. aerogenes is mostly linked to clinical settings and geographically widespread outbreaks (2). The emergence of ECC and K. aerogenes as potent nosocomial pathogens was further aggravated by the worldwide occurrence of multidrug resistant clones (5).

Hoffmann et al. separated the ECC into 12 (I to XII) diverse genetic clusters based on *hsp60* single marker sequencing (6). Currently, *hsp60* typing remains the most widely used method of categorizing species within the ECC (5). With the advent of whole-genome sequencing (WGS) however, average nucleotide identity (ANI) analysis is becoming the gold standard for *in silico* species assignment. Using such approaches on the entire collection of RefSeq Enterobacter genomes allowed the distinction of 18 phylogenetic clusters (A to R) within the ECC (7).

Carbapenems are first-line drugs for the treatment of extended-spectrum β-lactamase (ESBL) producing pathogens (8). ECC members were among the first *Enterobacteriaceae* to harbor carbapenem resistance determinants and are currently the second most prevalent carbapenem resistant *Enterobacteriaceae* (CRE) in the United States and other major regions (3). Carbapenemase producing Enterobacter spp. (CPE) isolates in hospital acquired infections (HAIs) predominately belonged to the ECC member *E. hormaechei* with its various respective subspecies (3, 9). *E. hormaechei* subsp. *xiangfangensis* (CIII) and *E. hormaechei* subsp. *steigerwaltii* (ST114, ST90 and ST93) are the most geographically prevalent CPEs (9).

There is a wide data schism regarding the epidemiology and dissemination of clinically significant K. aerogenes, and it is only lately that interest in this pathogen is rising. However, data related to the global burden of resistant subtypes of this pathogen are still scarce, especially in the Middle East (10). Well documented clinical cases of carbapenem resistant K. aerogenes (CR-KE) in the United States, Europe, and Asia were linked to plasmid encoding carbapenemases (2). Carbapenem resistance mechanisms in CR-KE were mainly linked to the constitutive overexpression of chromosomal AmpC along with changes in membrane permeability through porin regulation (10).

The ominous antimicrobial resistance (AMR) phenomenon has become a global issue in general and in Lebanon in particular (11). Various studies in Lebanon described alarming cases of carbapenem resistance in members of the family *Enterobacteriaceae*. Arabaghian et al. reported a 70.6% rate of total carbapenem resistance in a Klebsiella pneumoniae isolate set from a tertiary care hospital (12), and Alousi et al. reported the first ST-405 Escherichia coli single isolate carrying the *bla*_OXA-48_ carbapenem resistance determinant (13). Despite their notoriety as highly resistant nosocomial pathogens, members of Enterobacter spp. are not well studied. The sporadic studies that tackled their dissemination and resistance patterns were based on phenotypic screening and PCR assays (14, 15). A recently published comprehensive statistical report about ESBL and carbapenemase carriage in Lebanon for ESKAPE group pathogens also lacked detailed molecular characterization (16).

We aimed in this study to set up a molecular workflow for the accurate AMR detection and identity delineation of ECC and K. aerogenes isolates recovered from Lebanon. To that end, we performed a detailed molecular characterization of 50 multidrug resistant (MDR) and K. aerogenes clinical isolates. We used WGS to elucidate their correct nomenclature, clonal relatedness, and resistance/virulence profiles. The sequenced genomes were subjected to both intra- and interanalysis that allowed us to build an in-depth genomic understanding of ECC and K. aerogenes isolates recovered from Lebanon and to position them within a larger, more comprehensive global context. This study also included detailed analysis pertaining to the pathogenesis and dissemination of the reemerging pathogen K. aerogenes in Lebanon.

## RESULTS

### Characterization of the study isolates.

Isolates were collected over a period of five years (2013 to 2018) as part of routine microbiological screening at the American University of Beirut Medical Center (AUBMC). Selection criteria used were to include the isolates that were recovered from an infection sites/sources and were nonsusceptibility (intermediate/resistant) to one or more of the three clinically used carbapenems (meropenem, ertapenem and imipenem) or a susceptible phenotype to the clinically tested antibiotics. Most of the isolates (61%; *n* = 36/59) were resistant to the three tested carbapenems while 7% (4/59) of isolates showed nonsusceptibility to two tested carbapenems. Only 5% (3/59) were resistant to only one. Isolates KAM8/9 and ENM28/29 were, only, susceptible to imipenem, whereas KAM1, KAM4 and ENM47 were, only, susceptible to meropenem and imipenem. Additionally, more than 60% of the isolates showed resistance to other antibiotic classes; 75% (44/59) were resistant to gentamicin and 61% (36/59) to trimethoprim-sulfamethoxazole.

**FIG 1:**
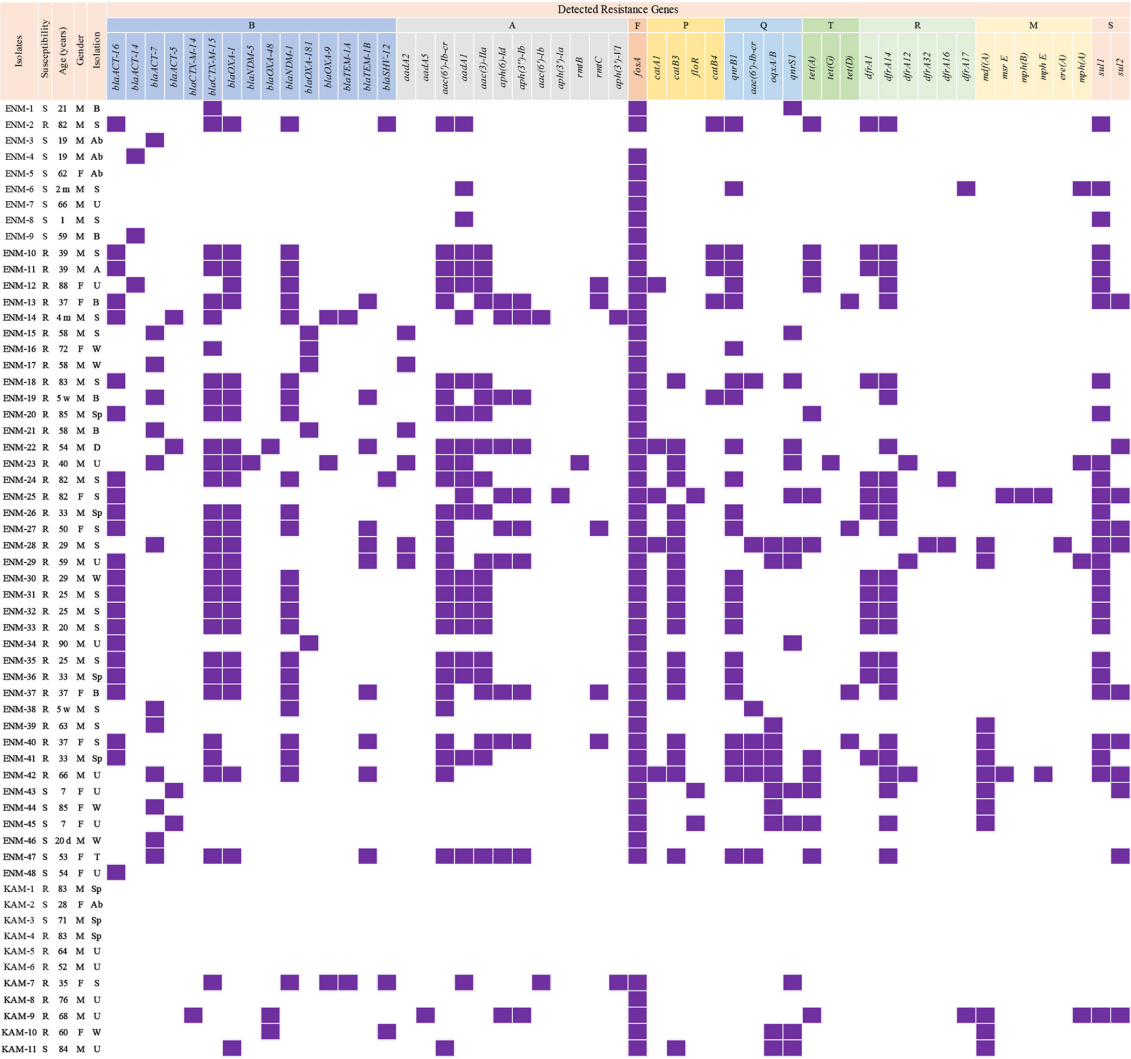
Isolates’ information and detected antibiotic resistance genes. In the susceptibility column, R: Resistant to Carbapenem; S: susceptible to Carbapenem. In the Age column, d: days m: months; w: weeks. In the gender column, F, Female; M, Male. In the specimen isolation column, A, Abscess; Ab, Abdominal fluid; B, Blood; D, DTA; S, Skin; Sp, Sputum; U, Urine; W, Wound.

ResFinder, used for the *in silico* analysis of antibiotic resistant determinants (ARD), uncovered the presence of 60 different unique resistance genes. The detected genes represented the nine clinically used classes and which included: β-lactams, aminoglycosides, fluroquinolones, trimethoprim, sulfonamide, fosfomycin, tetracyclines, macrolides and chloramphenicol (Fig. 1). Chromosomal *ampC* genes were detected in all the ECC isolates.

The prevalence of carbapenem resistance determinants (CRDs), shown in Fig. 1, was validated using *in silico* WGS analysis. *bla*_NDM-1_ was dominant and detected in 41% (24/59) of the isolates. Second-most dominant gene was the class D oxacillinase *bla*_OXA-181_ at 8% (5/59) followed closely by *bla*_OXA-48_ at 5% (3/59). *bla*_NDM-5_ was only detected in one ECC isolate (2%; ENM23). *bla*_ACT-16_ and *bla*_ACT-7_ were the most common *ampC* genes at 39% (23/59) and 22% (13/59), respectively. Among the genes conferring the ESBL phenotype, *bla*_CTX-M-15_ and *bla*_OXA-1_ were the most observed variants in this study, with *bla*_CTX-M-15_ being present in 49% (29/59) of isolates and *bla*_OXA-1_ in 41% (24/59), and their presence was not mutually exclusive. Seven isolates (KAM5, KAM6, KAM8, ENM25, ENM28/29 and ENM39) did not harbor any carbapenemase encoding gene. ENM-25 and ENM-39 only harbored their respective chromosomal *ampC* determinant, while ENM-28 and ENM-29 harbored ESBL encoding genes (Fig. 1).

Analysis of outer membrane protein-encoding genes against functional references (KT894106.1, AF336096.1, AF336095.1, AF335467.1) showed that only KAM-5 and KAM-6 had a mutated form of the *omp36* with a premature insertion of an Amber stop codon, while in the remaining six we didn’t detect any nonsynonymous mutation.

### *Hsp60* typing and average nucleotide identity analysis.

**FIG 2:**
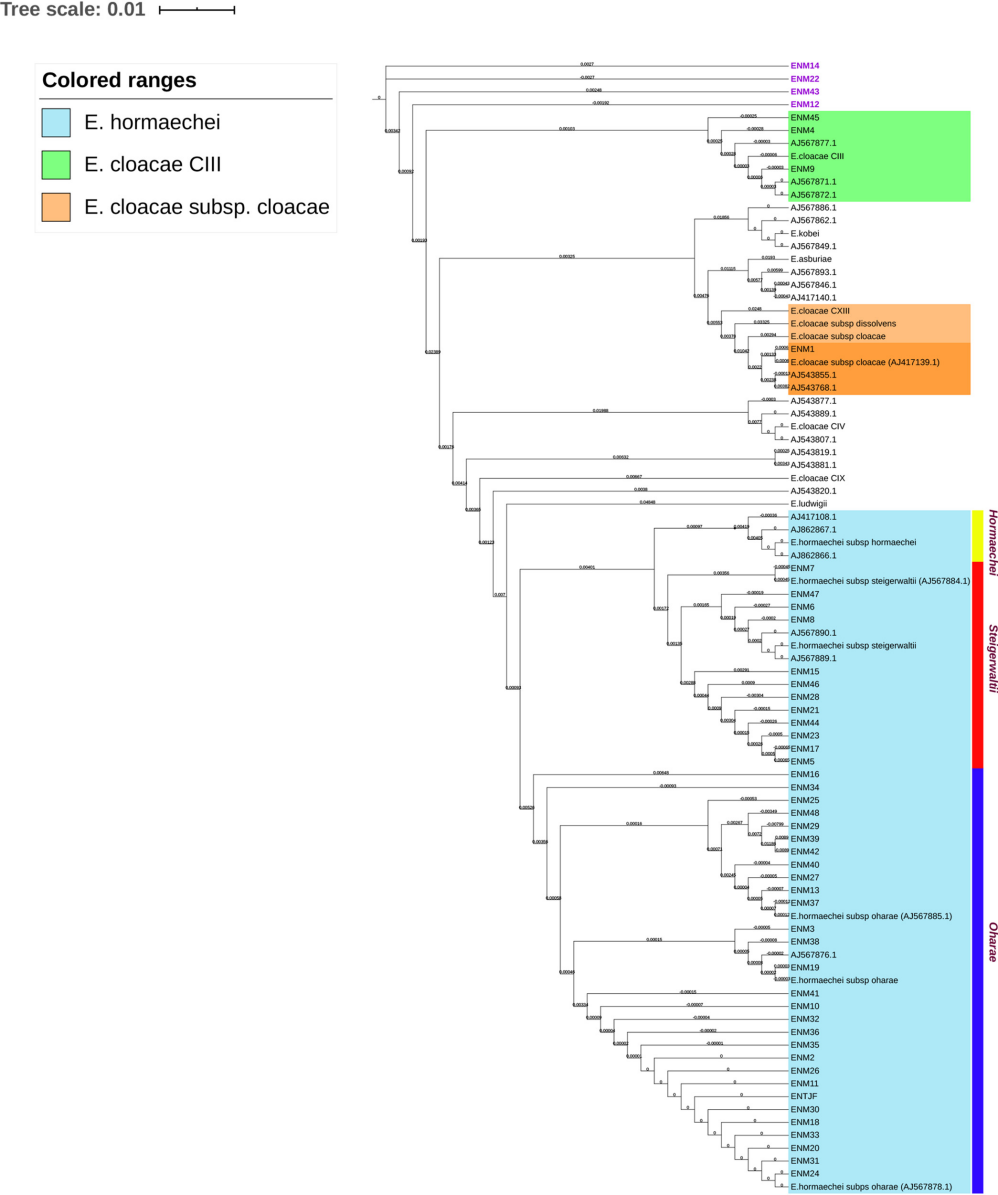
Neighbor-Joining (NJ) tree based on multisequence alignment of partial *hsp60* gene for all ECC isolates. Isolates clustering with reference *hsp60* genes were highlight within colored ranges. *Hormaechei* subspecies were demarcated through colored strips. Isolates ENM12-14-22-43 were highlighted in purple as each has clustered as a singleton on the NJ tree.

MALDI-TOF identification placed 48 (81%) of the isolates under the ECC. The *hsp60* sequences for the 48 ECC members were also determined and aligned with type strain sequences, from the NCBI gene database (AJ417141, AJ543870, AJ543878, AJ543807, AJ417110, AJ546761, AJ417134, AJ567879, AJ417115, and AJ862866). Seven (15%) of the ECC matched with the E. cloacae cluster III, and only to the E. cloacae subsp. *cloacae* mini clade (Fig. 2). It is noteworthy that 40 (84%) of the isolates clustered with *E. hormaechei* type strain sequences (AJ567884.1, AJ567885.1, AJ567878.1); none was typed as subsp. *hormaechei*, 12 typed as subsp. *steigerwaltii* (25%) and 28 as *oharae* (58%) (Fig. 2).

Average nucleotide identity (ANI) testing was employed as a confirmatory and higher sensitivity test for species and subspecies identification. Eight refseq complete genomes (Table S1) were used as references representing K. aerogenes, E. cloacae spp. *cloacae*/*dissolvens* and the five subsp. of *E. hormaechei* (*xiangfangensis*, *hoffmannii*, *oharae*, *steigerwaltii*, *hormaechei*). ANI values shown in Table S2, confirmed the identification of the 11 K. aerogenes. ECC isolates, however, revealed significant discrepancies between *hsp60* typing and ANI testing. According to the results of this study, the predominant *hormaechei* subsp. was *xiangfangensis* (54%; 26/48) followed by *steigerwaltii* (27%; 13/48) and *hoffmannii* (15%; 7/48). Only one *E. hormaechei* (ENM-48) belonged to the subsp. *oharae* and one was E. cloacae subsp. *cloaca* (ENM-1).

### Typing.

**FIG 3:**
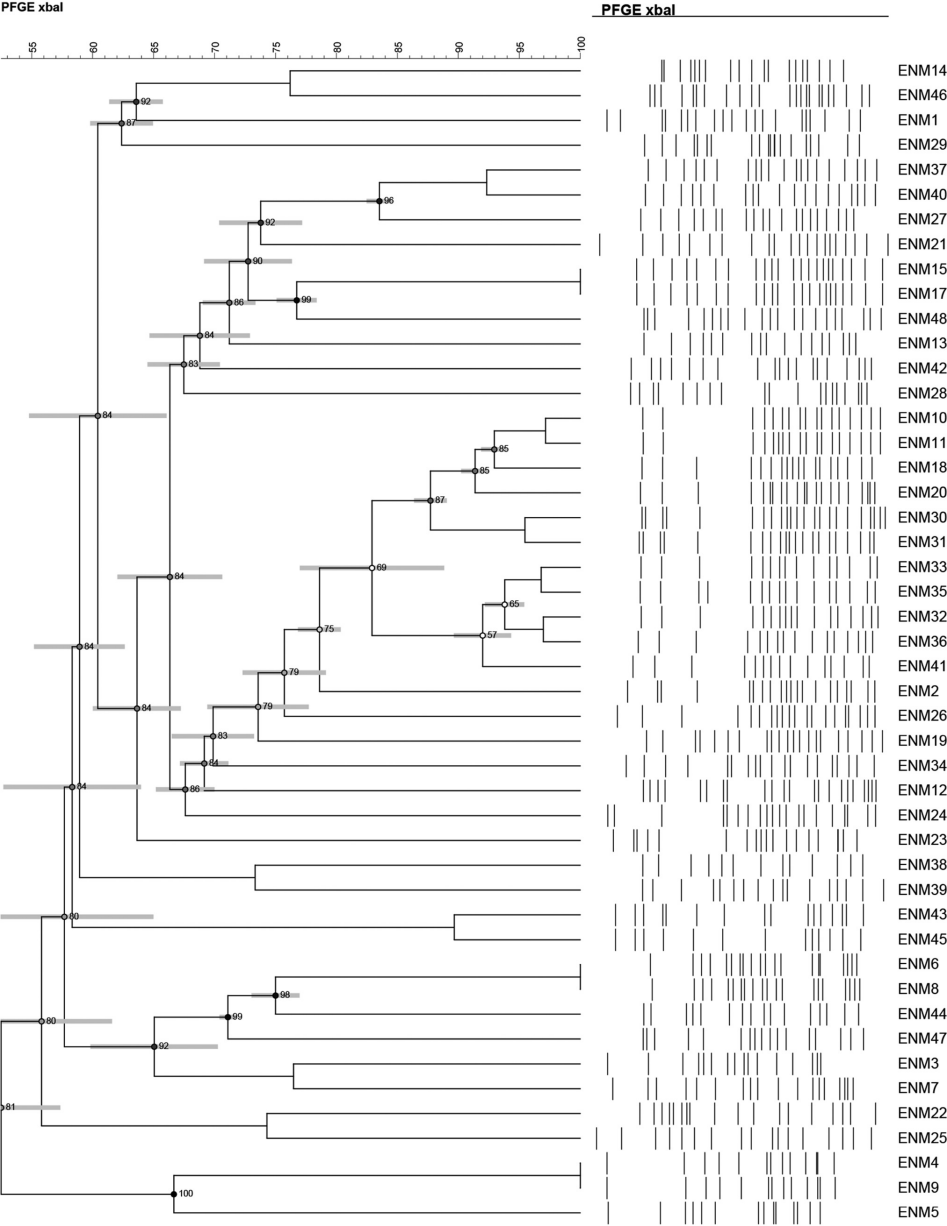
PFGE dendrogram of ECC isolates. Dendrogram was generated by BioNumerics software version 7.6.1 showing isolate clonality based on their banding patterns generated by XbaI restriction digestion. The results were inferred using the dice correlation with optimization and tolerance values set at 1.5%. Isolates’ STs and pulsotypes (PT) are also shown.

Twenty-four different sequence types (STs) were detected among the ECC, the most common were ST114 (29.2%; 14/48), ST182 (10.4%; 5/48), and ST190 (6.25%; 3/48). With K. aerogenes, seven distinct STs were detected, including: ST143 (18.2%; 2/11), ST93 (18.2%; 2/11), ST209 (18.2%; 2/11) and ST210 (18.2%; 2/11) (Fig. 3).

PFGE was performed to study the genetic relatedness between the isolates. A total of 36 distinct pulsotypes were identified. ENM-37 and ENM-40 showed a similarity of 93% based on the banding patterns (Fig. 3). These two isolates are of the same subsp. (*E. hormaechei* subsp. *xiangfangensis*) belonging to ST182, and with mostly identical resistance and plasmid profiles. ST114 isolates however, had seven different pulsotypes; PFGE banding patterns did not match fully with the MLST typing profiles. All ST114 isolates shared ≥80% PFGE similarity patterns and identical resistance profiles and determinants, except for ENM-24 and ENM-16 which, respectively, had a different pulsotype or was untypeable even after treatment with secondary enzyme.

**FIG 4:**
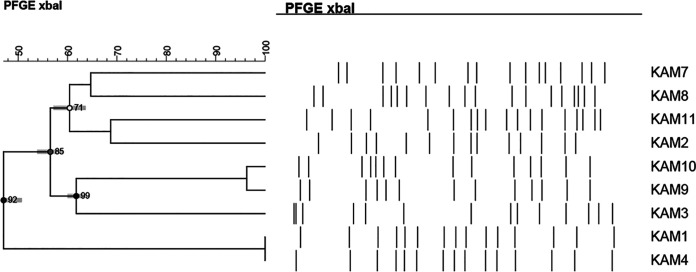
PFGE dendrogram of K. aerogenes isolates. Dendrogram was generated by BioNumerics software version 7.6.1 showing isolate clonality based on their banding patterns generated by XbaI restriction digestion. The results were inferred using the dice correlation with optimization and tolerance values set at 1.5%. Isolates’ STs and pulsotypes (PT) are also shown. Isolates KAM5 and KAM6 were untypeable.

Seven different pulsotypes were determined based on clustering and banding patterns for K. aerogenes (Fig. 4). Some of the banding patterns matched with the MLST; KAM-9 and KAM-10, typed as ST143, shared high clonal relatedness with only one band difference. KAM-2 and KAM-11 on the other hand, were typed as ST93 but had distinct banding patterns.

We used Kaptive, a web-based tool for the *in silico* k-type determination through targeting the complete *cps* locus, and detected three different K-types among the studied K. aerogenes isolates. All the isolates had the KL68 K-type except for KAM-3 and KAM-8, which, respectively, were identified as KL107 and KL26 (Table S3).

### Phylogenetic analysis.

**FIG 5:**
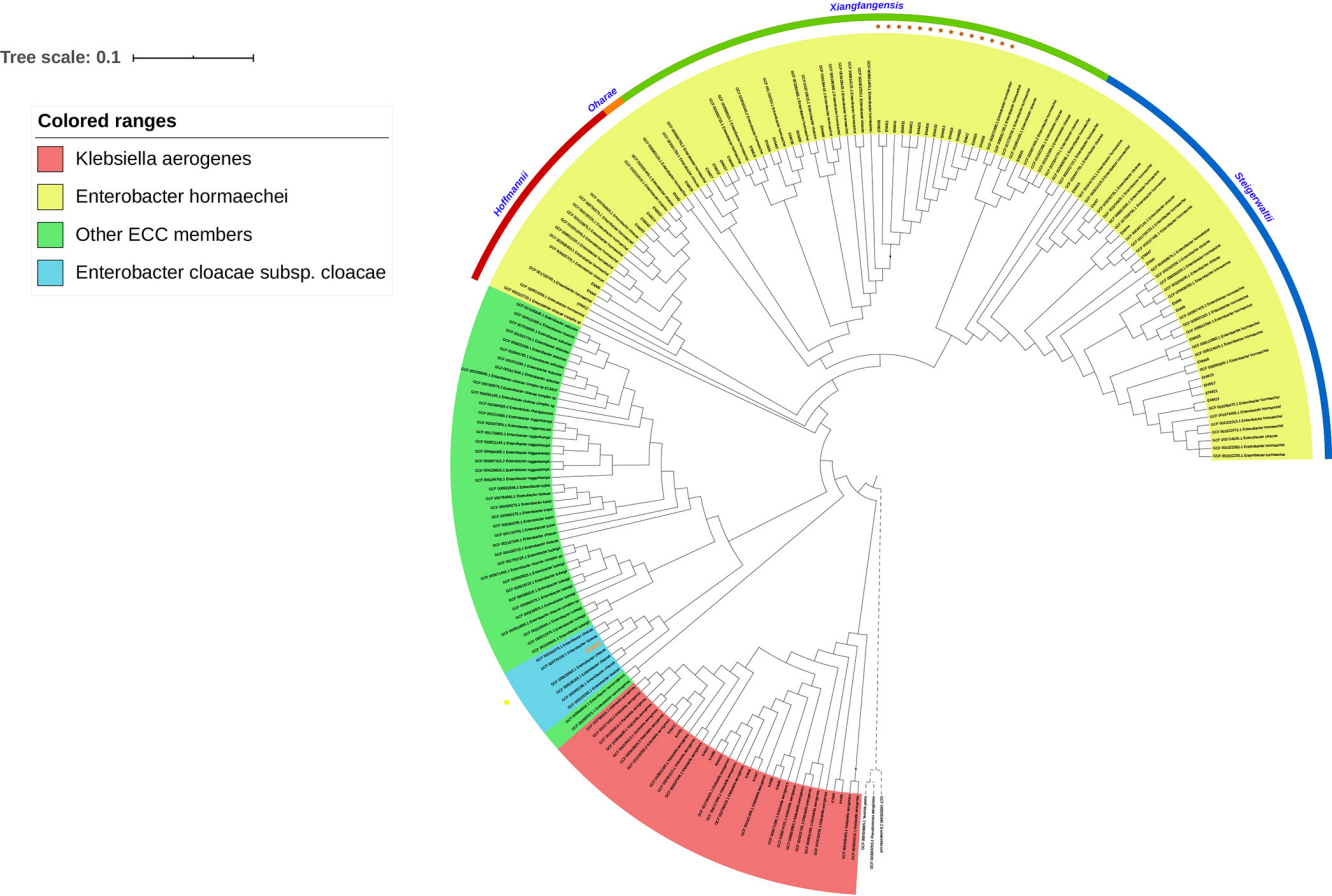
SCGs based maximum-likelihood phylogenetic tree of all study isolates and complete RefSeq ECC and K. aerogenes reference genomes. Colored ranges were used to differentiate between K. aerogenes and Enterobacter sp isolates. Subspecies of *E. hormaechei* are indicated by the outer rim-colored bands. Golden stars were used to highlight ST114 isolates, while the yellow dot was used to show the only E. cloacae subsp *cloacae* isolate (ENM1).

The primary phylogenetic placement of the isolates was performed using the GToTree pipeline. The analysis of 172 SCGs for all the isolates and complete RefSeq genomes clustered all into two major clades (I & II) (Fig. 5). Clade I (red block) distinctively harbored all the K. aerogenes (11/59) and at a large distance from the remaining Enterobacter spp. isolates (Fig. 5). All the ENM isolates (ENM1-48) clustered within clade II.

ECC members, except the *E. hormaechei* species, clustered in a subclade within clade II (green block) (Fig. 5). ENM1 (blue block) was the only isolate identified as closely related to the type strain E. cloacae subsp. *cloacae* ATCC13047 (CP001918.1). The remaining of ECC isolates were all grouped within one subclade (Fig. 5). Four *E. hormaechei* subsp. were detected, of which 26 (55%) belonged to the subsp. *xiangfangensis* and 14 (30%) were of ST114 (Fig. 5).

### Virulence of K. aerogenes.

We checked the virulence loci in K. aerogenes using Kleborate; a tool to screen for ICE*Kp* (virulence-associated integrative conjugative element of K. pneumoniae) associated virulence loci, including yersiniabactin (*ybt*), and colibactin (*clb*) (Table S3). The yersiniabactin locus (*ybtS*, *ybtX*, *ybtQ*, *ybtP*, *ybtA*, *ybtU*, *ybtT*, *ybtE*) encoding the yersiniabactin siderophore was detected in four isolates (36.4%; 4/11). KAM-2, KAM-3, and KAM-11 harbored the *ybt*17 on ICE*kp10* isoform, while in KAM-8 it was on a novel *ybt* locus variant. As for the bacterial toxin colibactin, all were negative except for KAM2, KAM3, and KAM-11, having the *clb3* colibactin locus.

### Plasmid identification and characterization.

**FIG 6:**
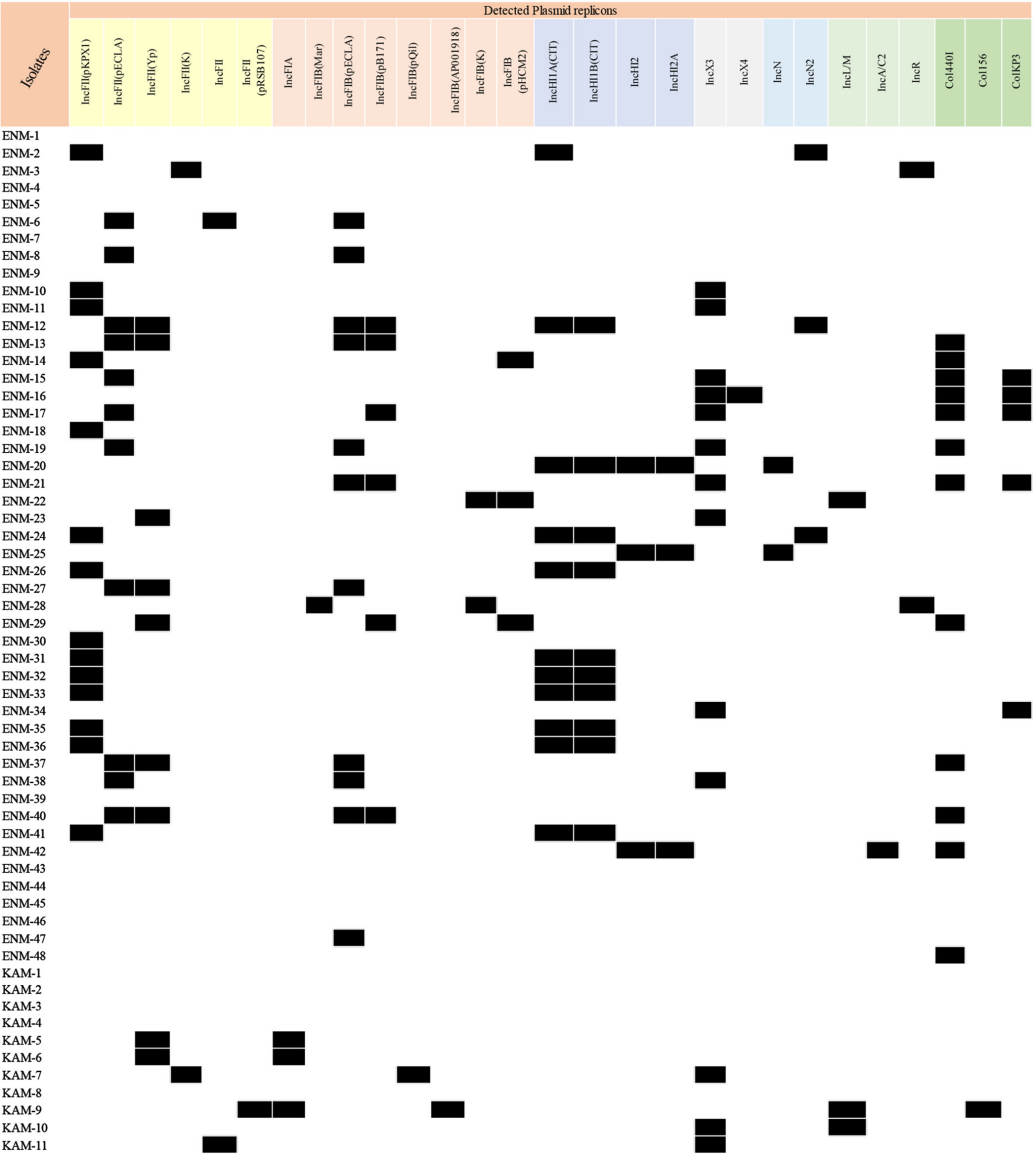
*In silico* detected plasmid incompatibility groups.

We used PlasmidFinder v2.1 and PBRT to identify the incompatibility groups for the recovered plasmids (Fig. 6). Distinct Inc groups were detected. IncFII was the most common (56%; 33/59) in the ECC and K. aerogenes, followed by IncFIB (33.9%; 20/59), IncX3 (22%; 13/59), IncHI1A (18.6%; 11/59), IncL (5.1%; 3/59), and IncA/C2 (1.7%; 1/59).

IncFII (pKPX1) was primarily associated with *bla*_NDM-1_, IncL with *bla*_OXA-48_ and was detected in three isolates (ENM-22, KAM-9, and KAM-10), and IncC was linked to *bla*_NDM-1_ positive isolate (ENM-42) lacking all IncF groups. All *bla*_OXA-181_ harboring isolates (ENM-15, ENM-16, ENM-17, ENM-21, and ENM-34) had the IncX3 plasmid in common and a single occurrence of *bla*_NDM-5_ was also linked to IncX3.

Three isolates, harboring the most prevalent CRDs, were selected for long read sequencing (ENM17, ENM30, and KAM9). We recovered three complete plasmids, which were identified and designated pLAU_ENM17_OXA181, pLAU_ENM30_NDM1 and pLAU_KAM9_OXA48.

Backbone analysis of pLAU_ENM17_OXA181 (MN792919) showed that it is an IncX3 self-conjugative plasmid, harboring a *bla*_OXA-181_ class D oxacillinase, with an overall size of 51,084 bp. The backbone components of pLAU_ENM17_OXA181 were consistent with a self-conjugative plasmid, harboring the necessary genes for propagation, stability, and replication. The *bla*_OXA-181_ was flanked upstream by an IS*Kpn19*/transposase, followed by a replicase. Downstream, the class D oxacillinase was flanked by three consecutive truncated transposases ΔIS*3000*/transposase-ΔIS*Ec63*-ΔIS*26*. The *qnrS1* fluoroquinolone resistance determinant was also identified on the plasmid and flanked upstream and downstream by IS*26* and Tn*3*-like resolvase, respectively. BLAST analysis showed 99% similarity between pLAU_ENM17_OXA181 and pSTIB_IncX3_OXA_181 (MG570092) isolated in Lebanon (17).

Based on the same analysis workflow, pLAU_KAM9_OXA48 was a 61,913 bp IncL conjugative plasmid. The IncL replicon identity was confirmed through PBRT and *in silico* BLAST analysis against publicly available IncL *repA* gene reference sequence in NCBI (NC_021488.1). Backbone screening of pLAU_KAM9_OXA48 identified conjugative transfer proteins (*tra* genes), *relB*/*relE* toxin-antitoxin system, and stability related proteins. *bla*_OXA-48_ was directly bracketed by a *lysR* gene upstream and a ΔIS*1R* downstream. However, analysis of the overall genetic environment identified two IS*10A* upstream and downstream of the class D oxacillinase as follows: IS*10A*-*lysR*-*bla*_OXA48_-ΔIS*1R*-IS*10A.* Interestingly, this cassette is like Tn*1999*, but has different insertion sequences bracketing the cassette. IS*10A* was found to be 99% identical to IS*1999* differing in the position of two SNPs. BLAST analysis of pLAU_KAM9_OXA48 also showed close relatedness to other plasmids.

pLAU_ENM30_NDM1 was linked to an IncFII plasmid through *in silico* scaffolding. Detailed analysis showed the presence of a copy of *bla*_NDM-1_ directly bracketed upstream by a ΔIS*Aba125* and downstream by a *ble*_MBL_ (bleomycin). Further analysis of *bla*_NDM-1_ genetic environment showed that it falls within a large genomic cassette harboring, from upstream to downstream: IS*3000*- ΔIS*Aba125*-*bla*_NDM-1_-*ble*_MBL_-//-Tn*5403*. BLAST analysis revealed high sequence similarity (>99%) between pLAU_ENM30_NDM1, pLAU_ENC1 (MN688131.1) (18), pAR_0128 (CP021720) and pKPX-1 (AP012055). Despite the high sequence similarity, there were size differences, with the latter three isolates larger than pLAU_ENM30_NDM1 by at least 90 Kbp.

## DISCUSSION

In this study, we performed an in-depth molecular characterization of 48 ECC and 11 K. aerogenes isolates, known previously as E. aerogenes, clinical isolates from Lebanon. We used molecular techniques backed by *in silico* whole-genome based characterization to resolve the molecular identity of the clinical isolates and determined and compared the phylogenetic relatedness, resistomes and virulomes. The distribution of resistance determinants was not uniform, the *bla*_NDM-1_ gene was commonly detected and associated with the IncF plasmid incompatibility group, class D oxacillinases, *bla*_OXA-48_ and *bla*_OXA-181_, were also prevalent and were associated with IncL and IncX3 plasmid types, respectively.

ECC members and K. aerogenes remain largely understudied and the focus often shifts toward E. coli and K. pneumoniae. Moghnieh et al. (2019) showed the antimicrobial susceptibility data gathered from 13 local hospitals in Lebanon. Data revealed the constant increase in carbapenemase and ESBL carriage among E. coli and K. pneumoniae (16). Moreover, a study in 2018 identified 27 carbapenem resistant E. coli clinical isolates in a Lebanese hospital, mediated by the dissemination of the class D oxacillinases *bla*_OXA-48_, *bla*_OXA-181_, and CTX-M-15 coupled with outer membrane porin alterations (19). Arabaghian et al. (2019) also characterized 34 multidrug resistant K. pneumoniae clinical isolates with all being at least resistant to ertapenem (12).

Our results illustrated a predominant presence of NDM-1 (41%; 24/59), especially within the ECC population, with three isolates harboring the *bla*_OXA-48_ two of which were identified as K. aerogenes (KAM9 and KAM10). Previous reports revealed that the Middle-East and the Mediterranean regions were endemic to *bla*_OXA-48_ (12, 38, 39), and which according to our results seem to be also attributed to the prevalence of K. aerogenes. *bla*_OXA-181_, a single base pair mutant analog of *bla*_OXA-48_, was also detected in 8% (5/59) of the isolates all of which were ECC members. Both NDM-1 and OXA-181 originated from the Indian subcontinent suggesting that the driving force for carbapenem resistance within ECC in Lebanon was similar to that seen in the Balkans and the South-Eastern regions of Asia (9, 20).

Our results also highlighted the extensive occurrence of CTX-M-15 (49%; 29/59), and its coexistence with other CRDs. This conforms with previous reports showing that CTX-M-15 was one of the most prevalent ESBLs within global members of the ECC (21, 22). Haenni et al. (2016) showed that CTX-M-15 positive high-risk E. cloacae clones in companion animals, namely, ST114, was alarmingly consistent (23). *bla*_CTX-M-15_ was mainly linked to mobile genetic elements but was chromosomal in ENM1. Analysis of its genetic environment revealed an IS*Ecp1* upstream and a disrupted ΔTn*2* downstream of *bla*_CTX-M-15_. In ENM1 the cassette was integrated downstream of the aerobactin operon, which could contribute to heightening the expression of virulence and resistance determinants during host infection (24–26).

ECC and K. aerogenes isolates are intrinsic carriers of chromosomal *ampC*. *ampC* is inducible and prone to acquire mutations leading to constitutive expression. When coupled with outer membrane porin alteration, primarily in K. aerogenes, carbapenamase independent resistance was observed (2, 4). KAM5 and KAM6 were devoid of any CRDs and displayed phenotypic carbapenem resistance. Analysis of *omp36* sequence revealed the presence of nonsynonymous point mutations (C→T), introducing a premature stop-codon. This is in line with the tendency of K. aerogenes to adapt when exposed to antimicrobial agents by inducing changes in porin permeability further complicating treatment (2, 10).

ST114 is an epidemic clone belonging to clonal complex CC114 linked to *bla*_CTX-M-15_ and recently to *bla*_NDM-1_ in Palestine, Italy, Japan, France, Spain, Greece, Latvia and other countries (9, 27). In our study, all ST114 isolates were NDM-1 positive highlighting the dissemination pattern of the carbapenem-resistant ECC from south-eastern Asia to Midwestern United States and the Middle East. On the other hand, ST78 MDR E. cloacae is a globally characterized ESBL clone harboring plasmidic *bla*_KPC_ (28). We detected two ST78 *E. hormaechei* subsp. *hoffmanni* isolates having *bla*_NDM-1_ or *bla*_OXA-48_ confirming the dissemination of high-risk clones within the ECC sublineages.

We detected yersiniabactin and colibactin siderophores in K. aerogenes. KAM-2, KAM-3, and KAM-11 had the conjugative and integrative ICE*Kp10* element. This element is associated with the mobilization of the yersiniabactin locus coharboring the colibactin (*clb3*) and yersinbactin (*ybt17*) siderophores (29). A diverse set of ICE*Kp* isoforms were detected in carbapenem-resistant K. pneumoniae isolated from clinical settings in Lebanon (12). None of the MDR isolates however, coharbored *clb* or *ybt*. The observed positive selection for K. aerogenes ICE*Kp* isoform suggested the selection of invasiveness (hypervirulence) over resistance (12, 29).

We identified three main CRDs, *bla*_NDM-1_, *bla*_OXA-48_ and *bla*_OXA-181_, on three separate plasmids. *bla*_OXA-48_ was detected in three isolates and associated with a 61,913bp self-conjugative plasmid pLAU_KAM9_OXA48. The plasmid being >99% similar to the OXA-48 reference plasmid pOXA-48a (JN626286.1) implicated its close relevance to the one originally recovered from K. pneumoniae isolated in Turkey in 2001 (30). Multiple studies reported *bla*_OXA-48_ dissemination locally and globally and its association with the ~60 Kbp widespread IncL plasmid (12, 13, 19, 31, 32). However, our study revealed a cassette-linked diversion. CRD on pLAU_KAM9_OXA48 was bracketed by IS*10A*, an isoform of IS*1999*.

OXA-181, a *bla*_OXA-48_-like variant first detected in New-Delhi in 2007, is currently considered the second most common *bla*_OXA-48_-like variant globally. The first report of *bla*_OXA-181_ in Lebanon was from an E. coli (17). pLAU_ENM17_OXA181 showed >99% similarity to pSTIB_IncX3_OXA181 (recovered from Lebanon; MG570092) and pOXA181 (KP400525.1) which showed that IncX3 was highly conserved despite its wide geographic spread (17, 30, 33).

Species and subsp. within the ECC are often incorrectly identified as E. cloacae (34, 35). In this study, only one E. cloacae (ENM1; 2%; 1/48) was detected while the remaining were identified as being *E. hormaechei* (98%; 47/48). The predominant, in accordance with previous reports (9, 36, 37), was *E. hormaechei* subsp. *xiangfangensis*.

To our knowledge, this is the first in-depth comprehensive whole-genome-based characterization of carbapenem-resistant ECC and K. aerogenes isolates in Lebanon and the region. In this study, genome-based workflows and molecular approaches were used to build a better understanding of the carbapenem-resistant populations recovered from clinical settings. Our results revealed that ST114, the epidemic global clone, was the predominant circulating clone (9). The prevalent ECC identified was *E. hormaechei* subsp. *xiangfangensis* and the *bla*_NDM-1_ was the most common carbapenemase. Collectively the correct identification and targeted WGS could help improve and monitor treatment approaches, mitigate outbreaks, and limit the spread, mobilization, and transmission of resistance determinants.

## MATERIALS AND METHODS

### Ethical approval.

Ethical approval for this project was not required as the study isolates were collected and stored as part of routine clinical screening. Patients’ records and information were anonymized, and clinical isolates data were deidentified before undertaking any form of analysis.

### Bacterial isolates.

A total of 59 ECC and K. aerogenes isolates were collected between 2013 and 2018 from the 350-bed tertiary care teaching hospital AUBMC as part of routine clinical care screening. Preliminary species identification was performed through the Matrix Assisted Laser Desorption/Ionization Time of Flight (MALDI-TOF) system (Bruker Daltonik, GmbH, Bremen, Germany) following the manufacturer’s instructions. Accordingly, 81% (48/59) were identified as members of the ECC, whereas 19% (11/59) belonged to the K. aerogenes species and were designated ENM1-48 and KAM1-11, respectively. Isolates were recovered from different infection sites/sources: skin, urine, wounds, and abdominal fluids. The mean patients’ age was 47 ± 26 years, and the range was 20 days-90 years.

### Antibiotic susceptibility testing.

Antibiotic susceptibility testing was performed through the Kirby-Bauer disk diffusion test (DDT) on Muller-Hinton agar, including 18 antibiotic disks representing eight categories of regularly used antimicrobial agents in clinical settings. The MIC was determined for eight selected antibiotics. All results were interpreted following CLSI recommendations (38, 39).

### Bacterial DNA extraction.

Colonies were grown overnight on tryptone soy agar (TSA) plates. The Nucleospin Tissue kit (Macherey-Nagel, Germany) was used for bacterial DNA extraction according to the manufacturer's instructions. The obtained DNA was used for downstream PCR assays and WGS.

### *Hsp60* gene amplification and sequencing.

PCR amplification and sequencing were performed with the *hsp60*-F and *hsp60*-R primers as previously described (6). *hsp60* sequences were aligned with references representing the 12 Hoffmann clusters (I→ XII) using Clustal-Omega alignment tool (https://www.ebi.ac.uk/Tools/msa/clustalo/) (40). The Hoffman cluster neighbor-joining (NJ) tree was visualized using the interactive tree of life (iTOL) tool (41).

### Pulse field gel electrophoresis (PFGE).

PFGE was carried out using XbaI restriction enzyme (ThermoScientific, Waltham, MA, USA), according to the PulseNet standard protocol (https://pulsenetinternational.org/; https://www.cdc.gov/pulsenet/). Fingerprint analysis was performed using BioNumerics software version 7.6.1 (Applied Maths, Sint-Martens-Latem, Belgium) and PFGE profiles were categorized as pulsotypes based on the criteria of a minimal difference of three or more bands between select isolates (42). Fingerprint clusters were inferred with the BioNumerics software based on the Dice correlation with optimization and tolerance values set at 1.5%.

### Multilocus sequence typing (MLST).

MLST was carried out for ECC members as previously described by Miyoshi-Akiyama et al. (43) and resulting sequences were curated versus the public database for ECC isolates (https://pubmlst.org/ecloacae/). K. aerogenes sequence types (ST) were also determined using the publicly available database for the molecular typing of K. aerogenes (https://pubmlst.org/kaerogenes/).

### Plasmid based replicon typing (PBRT).

Plasmid incompatibility (Inc) types were elucidated using the DIATHEVA PBRT kit (Diatheva, Fano, Italy) through a PCR-based replicon typing method consisting of eight multiplex PCR assays for the amplification of 25 replicons. All reactions were carried out according to the manufacturer’s instructions, including positive controls for all assays.

### Conjugative transfer.

The transfer of resistant markers (*bla*_NDM-1_, *bla*_NDM-5_, *bla*_OXA-48_, and *bla*_OXA-181_) was determined by conjugation using a plasmid-free recipient azide resistant E. coli J53 and the study isolates as donors. Single colonies of both the donor and recipient were inoculated on Luria-Bertani (LB) broth and incubated overnight at 37°C. Equal volumes of the donor and the recipient cultures were mixed and incubated overnight at 37°C, followed by plating on LB agar supplemented with meropenem. To ensure the effectiveness of the selected plates or the used antibiotics, positive and negative controls were run in parallel by separately plating the donor and recipient. Presence or absence of carbapenemases was determined through a PCR assay on the transconjugants followed by PBRT.

### Whole-genome sequencing and quality control (QC).

Genomic libraries of all 59 study isolates were created using the Nextera XT DNA library preparation kit with dual indexing (Illumina). The resulting libraries were sequenced on an Illumina MiSeq with forward and reverse reads of 300 bp length.

PacBio Sequel I long sequencing platform (Pacific Biosciences, Menlo Park, CA, USA) was also used for three representative isolates, ENM17, ENM30, and KAM9, as previously described (44). Quality assessment for the raw reads was done using FastQC version 0.11.8 and QUAST version 5.0.2 (45, 46). Adapter removal and trimming was performed through Trimmomatic v0.39 (47).

### Assembly, identification, annotation, and genome analysis.

Long read sequencing data were assembled using the HGAP4 De Novo Assembly Application (Pacific Biosciences) with a minimum seed coverage of 30×. Short read trimmed sequences were assembled using SPAdes version 3.14 with read error correction enabled (48).

Accurate species identification was performed through Average Nucleotide Identity (ANI) testing. FastANI tool was used to generate a distance matrix of ECC isolates against Refseq complete *E. hormaechei* and E. cloacae genomes (Table S1) (49). Subsequently, the assembled genomes were uploaded and annotated with the RASTtk (http://rast.nmpdr.org). ResFinder 4.1 (https://cge.food.dtu.dk/services/ResFinder/) and the comprehensive antibiotic resistance database (CARD) were used to assess resistance determinants in all the isolates (50, 51). Sequences of the *omp35* and *omp36* porin genes were extracted from the K. aerogenes genomes, and sequences were translated to detect nonsynonymous mutations using the translate webtool available at the Bioinformatics resource portal (https://web.expasy.org/translate/). Putative K-types and virulence determinants (yersiniabactin and colibactin) were inferred with the Kleborate and Kaptive (52).

### Plasmid studies.

PlasmidFinder 2.1 was used to detect and confirm the plasmid Inc types found in the study isolates (53). Potential plasmidic contigs resulting from long read sequencing were extracted and the closest matching reference was detected through the BLASTn tool on NCBI (www.ncbi.nlm.nih.gov/BLAST). PLACNETw was also used to identify plasmid sequences from short read sequencing data (54). Sequences were aligned and overlapping bases were trimmed and contiguous sequences were manually assembled. Open reading frames (ORFs) were inferred with Prodigal v2.6.1 with the closed option enabled (55). All coding sequences were investigated and manually annotated through a BLASTp strategy and the IS finder database for insertion sequences (https://isfinder.biotoul.fr/).

### Phylogenetic analysis.

The GToTree pipeline was used to determine the phylogenetic relationship between the study isolates (ECC and K. aerogenes). The pipeline relies on the analysis of 172 single-copy genes (SCGs) unique to *Gammaproteobacteria* and outputs a phylogenetic tree based on the comparison of the selected SCG set within the specified input files (56). All ECC and K. aerogenes complete Refseq genomes were downloaded from NCBI with the “complete genome” and “latest Refseq” filters. The final tree was generated and visualized on iTOL (41).

### Data availability.

All sequences were deposited at DDBJ/EMBL/GenBank under the BioProject number PRJNA551102. Plasmid sequences of pLAU_ENM30_NDM1, pLAU_ENM17_OXA181, and pLAU_KAM9_OXA48 were deposited at DDBJ/EMBL/GenBank under the following accession numbers: MN792917, MN792918, and MN792919.
